# Development of Duplex Loop-Mediated Isothermal Amplification with Hydroxynaphthol Blue for Detection of Infectious Spleen and Kidney Necrosis Virus and *Aeromonas hydrophila* in Chinese Perch (*Siniperca chuatsi*)

**DOI:** 10.3390/microorganisms13030586

**Published:** 2025-03-04

**Authors:** Xiao He, Jingyi Wu, Xu Tan, Sunan Xu, Weiguang Kong, Xiaodan Liu

**Affiliations:** 1 College of Animal Science and Technology, Yangzhou University, Yangzhou 225009, China; 15861361552@163.com (X.H.); 231903126@stu.yzu.edu.cn (J.W.); 231903121@stu.yzu.edu.cn (X.T.); 13295267288@163.com (S.X.); 2 Key Laboratory of Breeding Biotechnology and Sustainable Aquaculture, Institute of Hydrobiology, Chinese Academy of Sciences, Wuhan 430072, China; 3 International Research Laboratory of Prevention and Control of Important Animal Infectious Diseases and Zoonotic Diseases of Jiangsu Higher Education Institutions, Yangzhou University, Yangzhou 225012, China

**Keywords:** infectious spleen and kidney necrosis virus, *Aeromonas hydrophila*, visual duplex LAMP, detection

## Abstract

Bacterial sepsis caused by *Aeromonas hydrophila* (*A. hydrophila*) and infectious spleen and kidney necrosis virus disease (ISKNVD) caused by infectious spleen and kidney necrosis virus (ISKNV) frequently result in significant mortality among Chinese perch (*Siniperca chuatsi*). Co-infection of mandarin fish with *A. hydrophila* and ISKNV occurs from time to time. In this study, a visual detection method for ISKNV and *A. hydrophila* was developed, using loop-mediated isothermal amplification (LAMP) and pre-addition of hydroxynaphthol blue. Primers for amplifying LAMP in the same system were designed based on the conserved regions of the MCP gene of infectious spleen and kidney necrosis virus, as well as the *hlyA* gene of *A. hydrophila*. The results showed that this method amplified bright trapezoidal bands in the presence of only *A. hydrophila* or ISKNV and both, with sky blue for positive amplification and violet for negative amplification. There was no cross-reactivity with other pathogens, and fragments of 182 bp, 171 bp and 163 bp appeared after digestion of the *A. hydrophila* LAMP product and 136 bp, 117 bp and 96 bp appeared after digestion of the ISKNV LAMP product. This holds true even when both positive products are present simultaneously. The minimum detection limit of this method was 100 fg for *A. hydrophila* and 100 fg for ISKNV, and the minimum detection limit for the mixed template was 1 pg. Overall, this method has high sensitivity and specificity to rapidly detect and distinguish between the two pathogens.

## 1. Introduction

Chinese perch (*S. chuatsi*) is a highly valued species in the freshwater culture in China [1]. Along with the growing scale of the cultivation and the decrease in the water quality in the culture environment, the incidence of the disease is becoming more and more severe [2,3]. Currently, infectious spleen and kidney necrosis virus (ISKNV) and *A. hydrophila* have emerged as significant threats to the successful and healthy growth of the Chinese perch culture industry [4,5,6].

ISKNV is a representative species of the genus Megalocytivirus [7], which is enveloped and belongs to double-stranded DNA viruses with an icosahedral structure. According to the classification established by the International Committee on Taxonomy of Viruses (ICTV) (https://ictv.global/report/chapter/iridoviridae/iridoviridae/megalocytivirus (accessed on 26 February 2025)), the species previously designated as infectious spleen and kidney necrosis virus (ISKNV) has been reclassified under the genus Megalocytivirus and is now formally referred to as *Megalocytivirus pagrus 1*. However, as the species designation *Megalocytivirus pagrus 1* encompasses a broad range of viruses within the genus, the term infectious spleen and kidney necrosis virus (ISKNV) is retained in this study to ensure precise and unambiguous identification of the specific viral strain under investigation. ISKNV infects a wide range of species, including a wide range of marine fish species in addition to freshwater fish [8]. It can lead to a large number of deaths of juvenile mandarin fish, and the cumulative mortality rate is as high as 100% [9]. This virus can even infect over 50 different species of marine and freshwater fish species, such as *Micropterus salmoides*, *Danio rerio*, *Xiphophorus maculatus* and *Lates calcarifer*. It has caused widespread damage to the aquaculture industry in many countries [8,10,11,12].

*A. hydrophila* is a member of a genus *Aeromonas* in the *Aeromonadaceae* family [13], and it is one of the most common pathogens in Chinese freshwater culture. It is capable of infecting a wide range of aquatic animals and poses a significant threat to terrestrial animals as well, and clinical studies have revealed that *A. hydrophila* can cause infection in humans either independently or in conjunction with other harmful bacteria [14,15,16]. Nielsen et al. carried out bacteriological examination of dead fish in five aquaculture fisheries in Zhejiang Province and isolated 95 bacteria strains. Among them, 58% and 53% of *A. hydrophila* were detected in the samples from *Megalobrama amblycephala* and *Carassius auratus* farms, respectively [17]. These isolated *A. hydrophila* were responsible for the haemorrhagic septicaemia of *Megalobrama amblycephala* and *Carassius auratus*. It is reported that it can also infect *Pelteobagrus fulvidraco*, *Alosa sapidissima*, *Procambarus clarkii*, *Cherax quadricarinatus*, *Eriocheir sinensis*, etc., and cause massive death [18,19,20,21,22]. Classical isothermal nucleic acid amplification technologies, including nucleic acid sequence-based amplification (NASBA), strand displacement amplification (SDA) and rolling circle amplification (RCA), are all technologies that can effectively amplify nucleic acids under isothermal conditions without the need for multi-step thermal cycling. NASBA can use the RNA T7 polymerase promoter to directly amplify RNA without amplifying double-stranded DNA, but the reliability of the analysis cannot be guaranteed for low concentrations of nucleic acids [23]. SDA is based on the ability of restriction enzymes to nick on the unmodified strand of the half-modified DNA recognition site and the ability of the 5′–3′ exonuclease-deficient DNA polymerase to lengthen the 3′ end at the nick and replace the downstream strand to amplify nucleic acids, a technique limited by the fact that buffers can affect the specificity of amplification [24]. Although RCA solves the problem of intrinsic amplification efficiency, it requires an additional ligation process prior to amplification for specific target identification [25]. Loop-mediated isothermal amplification was designed by Notomi et al. and first used for virus detection [26]. This technology has the advantages of simplicity and high sensitivity. In this study, the conserved sequences of ISKNV MCP gene and *A. hydrophila hlyA* gene were selected to establish a duplex LAMP detection method, and the two pathogens were distinguished by endonuclease digestion. This method is particularly suitable for rapid on-site diagnosis of pathogens, and positive and negative results can be visually distinguished directly.

The objective of this article is to develop a technique that enables the quick and concurrent identification of *A. hydrophila* and ISKNV and to provide an effective tool for the prevention of them.

## 2. Materials and Methods

### 2.1. Sample Collection and Preparation of DNA Templates

The bacteria and viruses used in this study such as *Siniperca chuatsi* Rhabdovirus (SCRV), Cyprinid herpesvirus 2 (CyHV-2), Largemouth bass ranavirus (LMBV), *A. hydrophila*, *Edwardsiella tarda*, *Plesiomonas shigelloides*, *Aeromonas sobria*, *Aeromonas schubertii*, *Aeromonas veronii*, *Aeromonas caviae*, *Pseudomonas aeruginosa* and *Vibrio parahaemolyticus* were isolated and preserved by the authors’ laboratory. All pathogens are validated by molecular techniques such as PCR, etc. Bacterial DNA extraction was performed using the MolPure^®^ Bacterial DNA Kit (Yeasen, Shanghai, China) in accordance with the guidelines provided by the manufacturer. Briefly, centrifuge the bacterial culture medium, discard the supernatant, and add lysis buffer to lyse the bacteria. Then, add proteinase K (Yeasen, Shanghai, China) to hydrolyze histones bound to nucleic acids, allowing DNA to dissociate in the solution, and add RNase A (Yeasen, Shanghai, China) to remove residual RNA. Add isopropyl alcohol (Macklin, Shanghai, China)for extraction, then add the deprotein solution from the reagent kit to the adsorption column and centrifuge to remove the protein. After washing with a rinse solution mixed with ethanol absolute (Sinopharm Chemical Reagent Co., Ltd., Shanghai, China), dissolve and gather with ddH_2_O (Takara Biomedical Technology (Beijing) Co., Ltd., Beijing, China). To obtain nucleic acid material for the virus, virus DNA extraction is performed using the Tissue DNA Kit (Yeasen, Shanghai, China) in accordance with the guidelines provided by the manufacturer. Briefly, take an appropriate amount of tissue samples and add lysis buffer, sterile water and proteinase K. After cutting, digest in a water bath to release the viral DNA into the solution. Centrifuge the supernatant, and add lysis buffer for secondary lysis, followed by ethanol absolute. Transfer the mixture into the adsorption column of the reagent kit and centrifuge, then add the deproteinized solution. Wash with rinsing solution mixed with ethanol absolute, and gather with ddH_2_O.

### 2.2. Primer Design and Synthesis

The major capsid protein (MCP) is a crucial component of the ISKNV virus structure, which has a decisive impact on the integrity and functional performance of the virus. The proportion of MCP protein in the entire soluble protein group of the virus is as high as 90% [5]. Hemolysin is the main virulence factor of *A. hydrophila*, with hemolytic and intestinal toxicity. It is closely related to the virulence of *A. hydrophila* [27]. All the published ISKNV MCP and *A. hydrophila hlyA* genes were compared based on the NCBI database, and the conserved regions were selected as the target fragments. According to the principle of primer design, multiple groups of primers of MCP and *hlyA* gene for LAMP can be amplified by using Primer Explorer V5 online primer design website (http://primerexplorer.jp/lampv5e/index.html (accessed on 5 June 2024)) and Snapgene 6.0.2 software. This set of primers was designed using the SnapGene 6.0.2 software-assisted primer design program. The specificity of the primers was ensured by targeting a unique genomic region. Comparative analysis of nearly 30 *A. hydrophila* strains from the NCBI database confirmed that the primers did not amplify non-target fragments of ISKNV or *A. hydrophila* in mandarin fish. Perform multiple sequence alignment to identify highly conserved regions between different isolates. This method aims to minimize the impact of genetic variation and ensure that primers are effective in various pathogen strains. All the oligonucleotides were synthesized by Tsingke Biotech Co., Ltd. (Nanjing, China) and stored at −20 °C until use (Table 1 and Table 2).

### 2.3. LAMP for ISKNV and A. hydrophila with Pre-Additional Hydroxynapthol Blue

The experimental procedures strictly adhered to zoning principles, encompassing distinct reagent preparation, sample processing and amplification areas, with the use of disposable consumables to prevent cross-contamination. Negative and blank controls were included in each experimental batch to monitor potential contamination. A LAMP system was established using Bst II DNA Polymerase Large Fragment, MgSO_4_, 10× IsothermalAmp Buffer and dNTPs mix from Nanjing Vazyme Biotechnology Co., Ltd. (Nanjing, China). The total amount of the system is 25 μL, and the specific content of each component is shown in Table 3 and Table 4. The reaction solution is maintained at 65 °C for 60 min. The color change after the reaction can be directly observed. For comparison, the LAMP amplification was detected by 3% agarose gel electrophoresis.

### 2.4. Optimization of LAMP-HNB

The positive nucleic acid mixed with ISKNV and *A. hydrophila* was used as a template to establish an optimized reaction system. The content of MgSO_4_, dNTP, reaction temperature and reaction time were optimized. Four reaction systems were designed, including 1.6 μM for MCP inner primers (FIP/BIP), 0.2 μM for MCP outer primers (F3/B3), 1.6 μM for *hlyA* inner primers (FIP/BIP), 0.2 μM for *hlyA* outer primers (F3/B3), 10 × IsothermalAmp Buffer 2.5 μL, 8 U Bst II DNA Polymerase Large Fragment, 120 μM hydroxynaphthol blue (HNB, Macklin, Shanghai, China) and 1 μL of mixed positive template. The content of MgSO_4_ was set as 6 mM, 7 mM, 8 mM, 9 mM and 10 mM; the content of dNTP was set as 1.0 mM, 1.2 mM, 1.4 mM, 1.6 mM and 1.8 mM; the reaction temperature was set as 60 °C, 61 °C, 62 °C, 63 °C, 64 °C and 65 °C; and the duration of the reaction was established at 30 min, 40 min, 50 min and 60 min. A 3% agarose gel electrophoresis analysis was performed after the reaction.

### 2.5. Specificity of the Duplex LAMP-HNB

The specificity of the duplex LAMP-HNB was determined using DNA or cDNA of *Siniperca chuatsi* Rhabdovirus (SCRV), Cyprinid herpesvirus 2 (CyHV-2), Largemouth bass ranavirus (LMBV), White spot syndrome virus (WSSV), Spring Viraemia of Carp Virus (SVCV), *A. hydrophila*, *Edwardsiella tarda*, *Plesiomonas shigelloides*, etc. These pathogens are either prevalent in mandarin fish farming or closely associated with the pathogens investigated in this study. The reaction system with ddH_2_O as template was set as the negative control. Three reaction systems were set up as positive controls that ISKNV, *A. hydrophila* and the mixed templates of ISKNV and *A. hydrophila* were used as templates.

### 2.6. Identification of Duplex LAMP-HNB Reaction Products by Enzymatic Digestion

Sequence analysis showed the presence of a SacII digestion site in the LAMP amplification target sequences of ISKNV and *A. hydrophila*, so the endonuclease digestion reaction could be used to analyze the two LAMP amplification products easily. The 20 μL enzyme digestion reaction with SacII endonuclease was performed according to the manufacturer’s instructions: DNA, 1 μL; QuickCut SacII (Takara Biomedical Technology (Beijing) Co., Ltd., Beijing, China), 1 μL; 10× QuickCut Buffer, 2 μL; sterilized water, 16 μL. This was incubated at 37 °C for 20 min and the digested product analyzed by 3% agarose gel electrophoresis. If 182 bp, 171 bp and 163 bp fragments were found after enzymatic digestion, the pathogen was *A. hydrophila*. The pathogen is ISKNV if 136 bp, 117 bp and 96 bp fragments are present following enzymatic digestion. Both *A. hydrophila* and ISKNV coexist when the above 6 sizes of fragments occur.

### 2.7. Sensitivity of the Duplex LAMP-HNB

The positive nucleic acids of ISKNV and *A. hydrophila* were mixed in equal amounts and diluted 10-fold to make the nucleic acid content in the reaction system of 100 ng to 100 fg. Using each gradient as a template, the duplex LAMP-HNB established above was performed. The amplified products were analyzed by agarose gel electrophoresis and visually inspected for chromogenic visuals.

### 2.8. Clinical Tests of the Duplex LAMP-HNB

A total of 30 pre-stored fish tissue samples from our laboratory were utilized as clinical testing materials. These samples, which were pre-stored in our laboratory, were collected from various ponds across three breeding farms at multiple time points. The duplex LAMP-HNB was employed for aquatic samples, and its performance was compared with conventional PCR. PCR primers are the external primers (F3/B3) used to establish the lamp method in this article.

## 3. Results

### 3.1. LAMP of ISKNV and A. hydrophila with Pre-Additional Hydroxynapthol Blue

**Figure 1:**
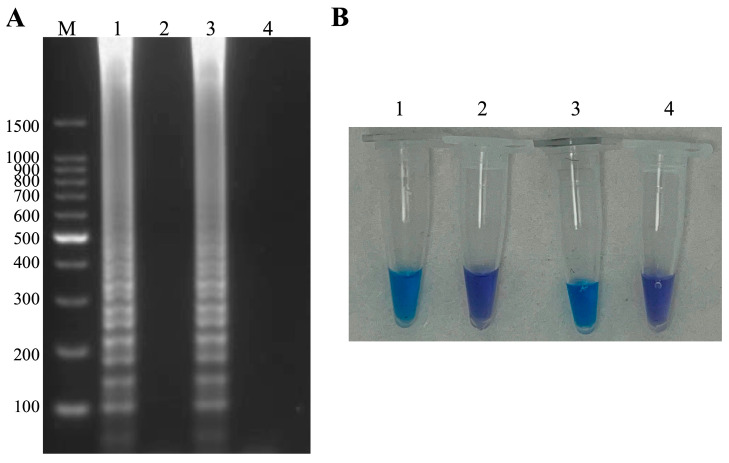
LAMP amplification of ISKNV and *A. hydrophila*, respectively. (**A**) M: 100 bp Ladder, 1–4 in that order: ISKNV, negative control, *A. hydrophila*, negative control. (**B**) Visual inspection: 1–4 in that order: ISKNV, negative control, *A. hydrophila*, negative control.

The LAMP product pre-supplemented with hydroxynaphthol blue showed an obvious ladder-like pattern using 3% agarose gel electrophoresis analysis (Figure 1A), and the results were consistent with the visual inspection results (Figure 1B). The amplification system of ISKNV and *A. hydrophila* was determined to contain 1.6 μM for FIP and BIP, 0.2 μM for F3 and B3, 2.5 μL for 10 × IsothermalAmp Buffer, 6 mM MgSO_4_, 1.4 mM dNTPs mix, 8 U Bst II DNA Polymerase Large Fragment, 120 μM hydroxynaphthol blue, 1 μL of DNA template and 11.7 μL of ddH_2_O.

### 3.2. Establishment of ISKNV and A. hydrophila Duplex LAMP-HNB

**Figure 2:**
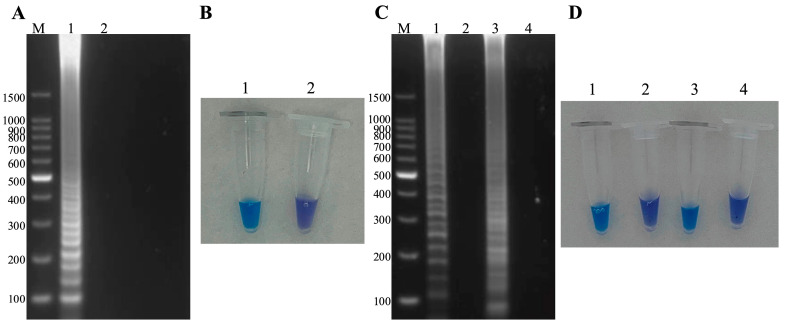
Development of the duplex LAMP-HNB detection method. (**A**) Dual-pathogen amplification: M: 100 bp Ladder, 1: ISKNV and *A. hydrophila*, 2: negative control. (**B**) Visual inspection: 1: ISKNV and *A. hydrophila*, 2: negative control. (**C**) Single-pathogen amplification: M: 100 bp Ladder, 1–4 in that order: ISKNV, negative control, *A. hydrophila*, negative control. (**D**) Visual inspection: 1–4 in that order: ISKNV, negative control, *A. hydrophila*, negative control.

After optimizing the Mg^2+^ content (Figure S1A), dNTP content (Figure S1B), reaction temperature (Figure S1C) and reaction time (Figure S1D), the optimal conditions were selected to establish the duplex LAMP-HNB. The results showed that optimal content of MgSO_4_ is 6 mM (Figure S1A line 1), optimal content of dNTP content is 1.6 Mm (Figure S1B line 4), optimal reaction temperature is 65 °C (Figure S1C line 6) and optimal reaction time is 60 min (Figure S1D line 4). The amplified bands under the above optimal conditions are brighter and clearer. In summary, the duplex LAMP-HNB reaction system is determined as MCP FIP 1.6 μM, MCP BIP 1.6 μM, MCP F3 0.2 μM, MCP B3 0.2 μM, *hlyA* FIP 1.6 μM, *hlyA* BIP 1.6 μM, *hlyA* F3 0.2 μM, *hlyA* B3 0.2 μM, 6 mM MgSO_4_, 1.6 mM dNTPs mix, 10 × IsothermalAmp Buffer 2.5 μL, 8 U Bst II DNA Polymerase Large Fragment, 120 μM hydroxynaphthol blue (HNB), 1 μL mixed positive template and 9.4 μL of ddH_2_O, incubated at 65 °C for 60 min. The method was able to amplify successfully in the presence of both dual and single pathogens (Figure 2A,C), and the visual results were consistent with the amplification results (Figure 2B,D).

### 3.3. Specificity Tests of the Duplex LAMP-HNB

**Figure 3:**
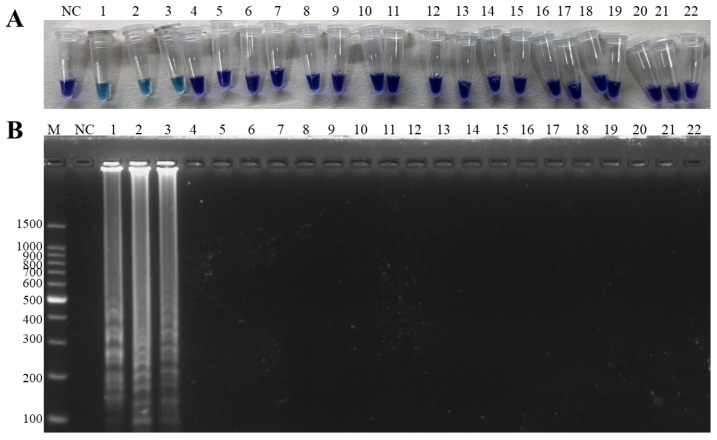
Specificity of the duplex LAMP-HNB. (**A**) Visual inspection: NC: negative control, 1–22 in that order: ISKNV, *A. hydrophila*, ISKNV and *A. hydrophila*, SCRV, CyHV-2, LMBV, WSSV, SVCV, *A. hydrophila*, *Edwardsiella tarda*, *Plesiomonas shigelloides*, *Aeromonas sobria*, *Aeromonas schubertii*, *Aeromonas veronii*, *Aeromonas caviae*, *Aeromonas salmonicida*, *Aeromonas jandaei*, *Pseudomonas aeruginosa*, *Pseudomonas fluorescens*, *Vibrio parahaemolyticus*, *Vibrio cholerae, Escherichia coli*. (**B**) M: 100 bp Ladder, NC: negative control, 1–22 in that order: ISKNV, *A. hydrophila*, ISKNV and *A. hydrophila*, SCRV, CyHV-2, LMBV, WSSV, SVCV, *A. hydrophila*, *Edwardsiella tarda*, *Plesiomonas shigelloides*, *Aeromonas sobria*, *Aeromonas schubertii*, *Aeromonas veronii*, *Aeromonas caviae*, *Aeromonas salmonicida*, *Aeromonas jandaei*, *Pseudomonas aeruginosa*, *Pseudomonas fluorescens*, *Vibrio parahaemolyticus*, *Vibrio cholerae*, *Escherichia coli*.

The specificity results showed that only ISKNV, *A. hydrophila* and hybrid templates amplified obvious bands, and the visual results were consistent with the electrophoresis results (Figure 3).

### 3.4. Identification of Duplex LAMP-HNB Enzymatic Digestion Products

**Figure 4:**
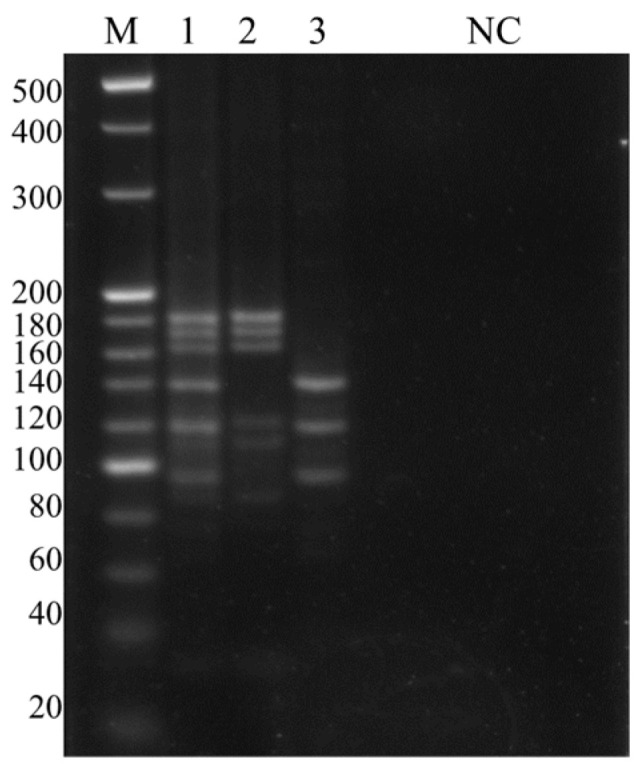
Identification of double LAMP by restriction endonuclease digestion. M: 20 bp Ladder, 1–3 in that order: *A. hydrophila* and ISKNV, *A. hydrophila*, ISKNV, NC: negative control.

Fragments of 182 bp, 171 bp and 163 bp appeared after digestion of the *A. hydrophila* LAMP product, and 136 bp, 117 bp and 96 bp appeared after digestion of the ISKNV LAMP product. The bands of corresponding sizes were also found after digestion of the duplex LAMP product of the mixed template (Figure 4). As shown in the inventor’s result, there also have some meaningless bands after digestion, and we suspect that the short cyclic structural chains during the amplification process are digested [26].

### 3.5. Sensitivity Tests of the Duplex LAMP-HNB

**Figure 5:**
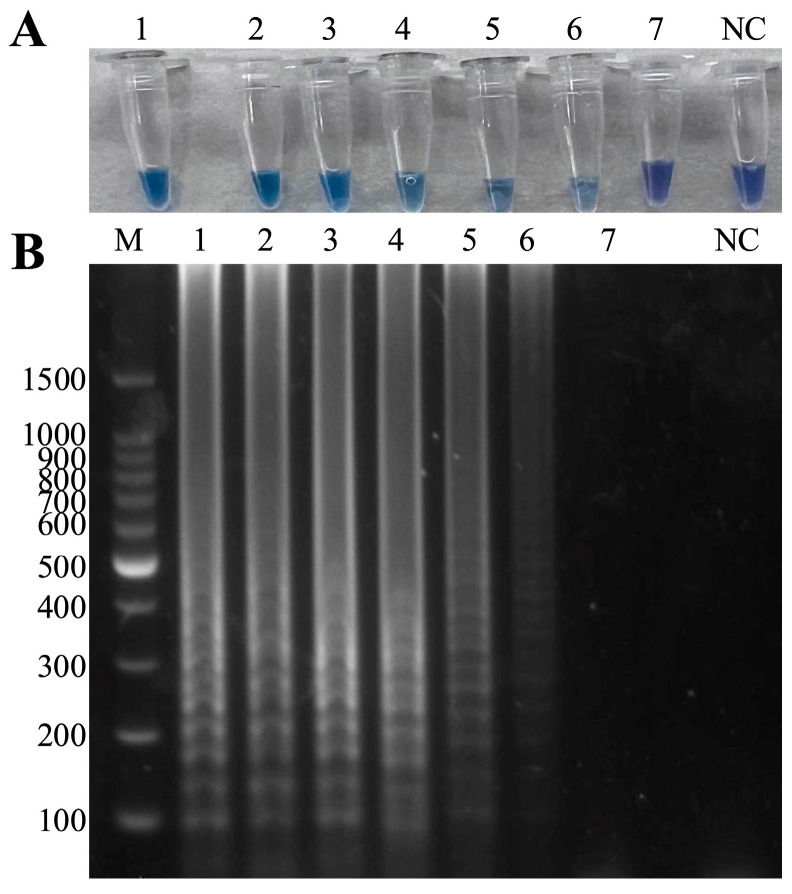
Sensitivity of the duplex LAMP-HNB for *A. hydrophila*. (**A**) Visual inspection: 1–7 in that order: 10 ng, 1 ng, 100 pg, 10 pg, 1 pg, 100 fg, 10 fg, NC: negative control. (**B**) M: 100 bp DNA Ladder, 1–7 in that order: 10 ng, 1 ng, 100 pg, 10 pg, 1 pg, 100 fg, 10 fg, NC: negative control.

**Figure 6:**
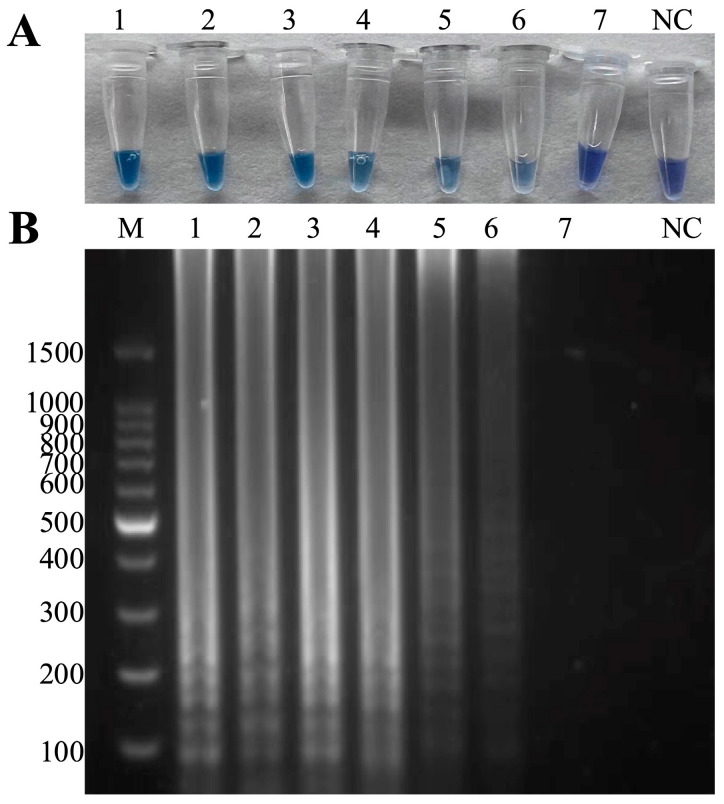
Sensitivity of the duplex LAMP-HNB for ISKNV. (**A**) 1–7 in that order: 10 ng, 1 ng, 100 pg, 10 pg, 1 pg, 100 fg, 10 fg, NC: negative control. (**B**) M: 100 bp DNA Ladder, 1–7 in that order: 10 ng, 1 ng, 100 pg, 10 pg, 1 pg, 100 fg, 10 fg, NC: negative control.

The minimum detection limit of this method was 100 fg for *A. hydrophila* (Figure 5B) and 100 fg for ISKNV (Figure 6B), and the visual results were consistent with the results of agarose gel electrophoresis (Figure 5A and Figure 6A).

**Figure 7:**
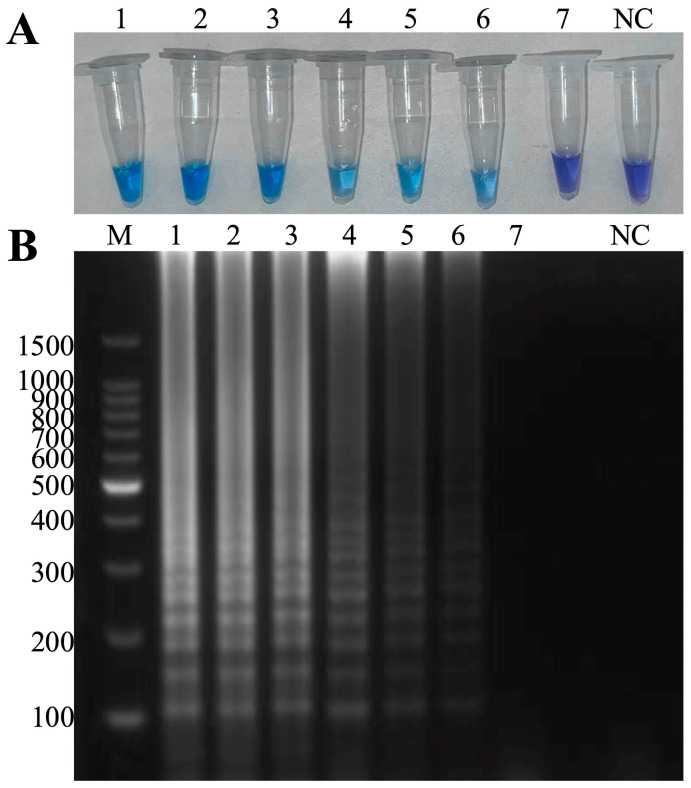
Sensitivity of the duplex LAMP-HNB. (**A**) 1–7 in that order: 100 ng, 10 ng, 1 ng, 100 pg, 10 pg, 1 pg, 100 fg, NC: negative control. (**B**) M: 100 bp Ladder, 1–7 in that order: 100 ng, 10 ng, 1 ng, 100 pg, 10 pg, 1 pg, 100 fg, NC: negative control.

The minimum detection limit for the mixed template was 1 pg, and the visual results were consistent with the results of agarose gel electrophoresis (Figure 7). The LAMP product with the lowest detection limit is enzymatically digested, and the electrophoresis product can also verify that the lowest detection limit can still detect *A. hydrophila* and ISKNV at the same time (Figure S2).

### 3.6. Comparison of the Duplex LAMP-HNB and Conventional PCR

**Figure 8:**
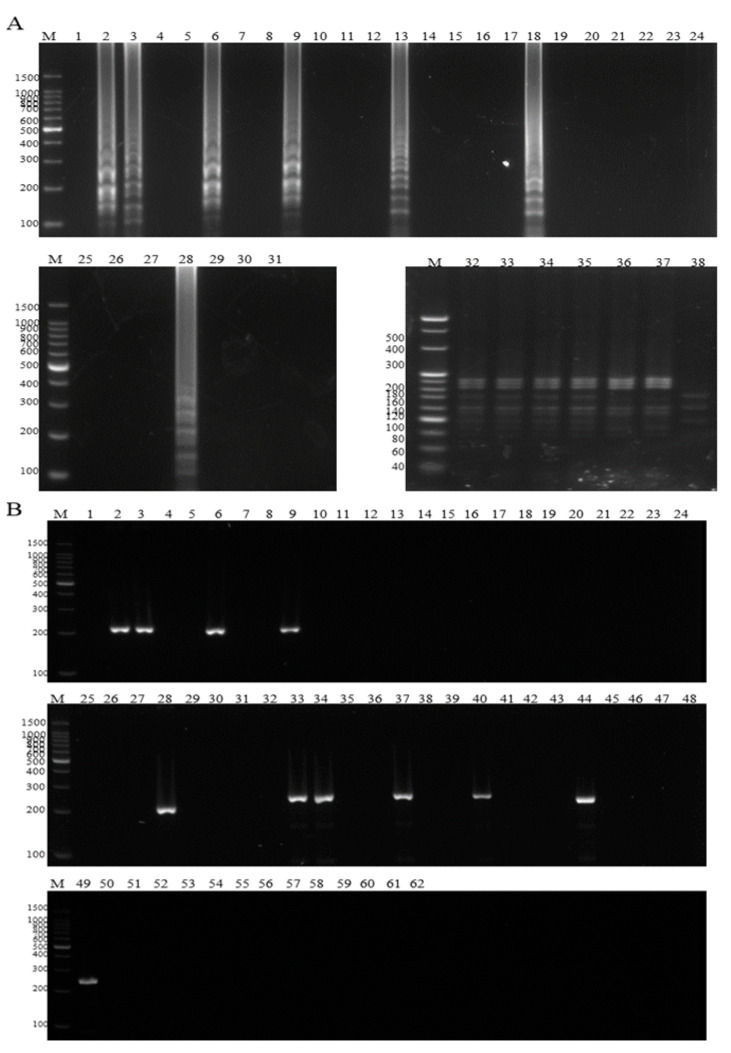
Comparison of the duplex LAMP-HNB and conventional PCR. (**A**) M: 100 bp DNA Ladder, M: 20 bp DNA Ladder, 1–30 in that order: 30 samples detected by duplex LAMP-HNB, 31: negative control, 32–38 in that order: corresponding to the enzyme digestion results of positive samples. (**B**) M: 100 bp Ladder, 1–30 in that order: 30 samples detected by duplex PCR for ISKNV, 31: negative control, 32–61 in that order: 30 samples detected by duplex PCR for *A. hydrophila*, 62: negative control.

Analysis of the test results revealed that seven of the thirty samples were positive, including four samples with dual-pathogen infections, one sample infected with ISKNV and two samples infected with *A. hydrophila* (Figure 8). Following enzymatic digestion of the product, the results were found to be consistent with those obtained from traditional PCR detection, demonstrating that the method developed in this study exhibits reliable performance for on-site detection (Figure 8).

## 4. Discussion

The LAMP technology is a nucleic acid amplification technology that can efficiently, rapidly and specifically amplify the DNA fragments of interest with a group of four specific primers under isothermal conditions and accumulate 10^9^ target copies in less than one hour [26]. Currently, LAMP has been widely applied to detect pathogens in aquaculture, such as parasites, bacteria and viruses [28,29,30]. Although LAMP offers the advantages of high sensitivity and rapid amplification, it is highly susceptible to residual contamination, which can lead to false-positive results. To address this issue, stringent zoning protocols and multiple negative controls were implemented in this study to minimize the risk of contamination. *A. hydrophila* and ISKNV are important pathogens in mandarin fish and can cause bacterial sepsis and infectious spleen kidney necrosis virus disease [31,32]. With the development of Chinese perch culture, co-infection of *A. hydrophila* and ISKNV has often become observed [33]. Under mixed infection conditions, mandarin fish exhibited severe clinical symptoms and notable histopathological alterations. The co-infection resulted in darkened body coloration, impaired swimming capacity and extensive hemorrhaging in tissues, including the gill filaments, fin base, liver, spleen and kidneys, in diseased individuals [34]. The interaction between *A. hydrophila* and ISKNV exhibits complexity under different co-infection modes, characterized by either antagonistic or synergistic effects. Overall, co-infection results in more severe clinical symptoms and higher cumulative mortality compared to single-pathogen infection. In mixed infections, several immune-related genes show elevated expression levels in the head and kidney tissues of mandarin fish. The mortality rate of mandarin fish infected with ISKNV 1–14 days post-secondary infection with *A. hydrophila* is significantly higher than that of the ISKNV-only group [34]. Consequently, controlling secondary bacterial infections may serve as an effective strategy to mitigate the overall mortality rate of hemorrhagic diseases in aquaculture practices. Therefore, there is a need for a rapid, specific, sensitive and cost-effective diagnostic method that can be used effectively for the simultaneous detection of *A. hydrophila* and ISKNV.

The pathogenic potential of *A. hydrophila* is attributed to the production of a diverse array of virulence factors. Among these, hemolysin (*hlyA*), aerolysin (*aerA*), cytotoxic enterotoxin (*act*), heat-labile cytotonic enterotoxin (*alt*) and heat-stable cytotonic enterotoxin (*ast*) are well-characterized virulence determinants. The *hlyA* is a hemolysin gene commonly found in *Aeromonas* species and genetically present within *A. hydrophila* [35]. In the study by Lian et al., it was also demonstrated that the gene exhibits a high degree of conservation among *A. hydrophila* species [36]. The ISKNV MCP gene sequence is highly conserved across members of the *Iridoviridae* family and serves as a reliable marker for distinguishing closely related iridovirus isolates [37,38,39]. As demonstrated in the study by Subramaniam et al., the MCP gene exhibits a high degree of conservation among diverse ISKNV strains from various geographical regions [40].

In this paper, we successfully established the first visual duplex LAMP-HNB method for the detection of *hlyA* gene of *A. hydrophila* and MCP gene of infectious spleen and kidney necrosis virus. The new duplex LAMP-HNB can detect both *A. hydrophila* and ISKNV in an isothermal step, followed by enzymatic analysis to distinguish between the two pathogens. In addition, a closed-tube LAMP pre-supplemented with HNB allows for visual analysis of the results during on-site testing to save time and avoid false positive contamination that may be caused by opening the tube and adding indicators [41,42]. Although the pre-addition of HNB eliminates the risk of contamination associated with opening reaction tubes [42], it remains unclear whether this approach offers superior contamination control compared to traditional LAMP methods, necessitating further investigation. The optimal content of MgSO_4_ is 6 mM (Figure S1A line 1), the optimal content of dNTP content is 1.6 Mm (Figure S1B line 4), the optimal amplification temperature of the Duplex LAMP-HNB was 65 °C and the optimal reaction time was 60 min (Figure S1C,D). Our method is designed for production-line detection, utilizing DNA extracted from fish tissue as the template. As demonstrated in Figure 8 of this study, the detection method is effective for complex matrices such as fish tissue DNA. Furthermore, it is potentially applicable to other scenarios involving purified viral DNA or bacterial genomic DNA. Given that fish tissue DNA represents a more complex matrix, the method is expected to perform effectively with simpler matrices, as detailed in subsequent analyses. The detection speed of the duplex LAMP-HNB method is similar to that of the traditional LAMP method, while the duplex LAMP-HNB method enables multiplexing in a single reaction.

The sensitivity of the duplex LAMP-HNB method was measured using serial dilutions of the genomic DNA template. The duplex LAMP-HNB method enabled minimal detection of 100 fg for *A. hydrophila* (Figure 5 and 100 fg for ISKNV (Figure 6), and the minimum detection limit for the mixed template was 1 pg (Figure 7). The sensitivity of this method is better than that Gao Z. et al. established for the detection of *A. hydrophila*, with a minimum detection limit of 0.559 ng/μL; the article also lists a qPCR assay with a minimum detection limit of 4.301 ng/μL [43]. However, the sensitivity was slightly inferior to the LAMP method published by Subramaniam K. et al. which had a minimum detection limit of 20 fg for ISKNV [37]. Compared with the LAMP method for the simultaneous detection of *A. hydrophila*, the duplex LAMP-HNB method established in this paper still has advantages in the sensitivity of the method [44]. The detection method developed in this study is capable of effectively identifying low-level infections of *A. hydrophila* and ISKNV, facilitating the recognition of early or subclinical infections, enabling early warning for disease prevention and control and mitigating risks in aquaculture practices.

PCR-based detection techniques for *A. hydrophila* and ISKNV have also been reported [45,46]. The detection method developed by Lin et al., based on dualplex RAA and CRISPR/Cas12a for pathogenic *A. hydrophila*, demonstrates high sensitivity, with a minimum detection limit of two copies of genomic DNA. However, the interpretation and differentiation of its results depend on the use of precision instruments. Additionally, the study by Lim et al. found that the LAMP-based detection method was five times more sensitive than the PCR-based detection method [47], and in terms of comparative detection specificity, the detection method based on the LAMP method performed better [48]. The TaqMan PCR assay established by Trakhna F. et al. had a detection limit of 1 pg for DNA from *A. hydrophila,* but it is limited by the reliance on precision instruments [49]. Similarly, the TaqMan real-time PCR developed by Lin et al. for detecting ISKNV is 10,000 times more sensitive than traditional PCR, but it is also limited by precise temperature instruments [50]. The sensitivity of the method in this article is not as high as these methods, but the advantage lies in their convenience, speed and low requirements, and compared with the sensitivity of some PCR detection methods, the method in this article has better sensitivity [51].

Other methods used in the laboratory for the detection of *A. hydrophila* and ISKNV include LAMP-based detection technology combined with microfluidic chips, lateral flow dipstick, etc., all of which have excellent sensitivity [52,53]. The dual-sample microfluidic chip detection method based on loop-mediated isothermal amplification established by Zhou Q J et al. can achieve a minimum detection limit of 10^–4^–10^–5^ pg/μL for ISKNV. This phenomenon can be attributed to the utilization of additional components, which enhance the efficiency of the LAMP reaction, and its template preparation is more refined. The interpretation of the results relies on the capture of fluorescent signals, which enables more accurate outcomes. However, the method using a fluorogenic loop-mediated isothermal amplification-based dual-sample microfluidic chip requires a fluorescence imaging system to acquire the endpoint and/or real-time fluorescent signals, which are displayed on the computer [44]. The cost associated with this approach is significantly higher compared to the method proposed in this study. The LAMP-LFD detection method established by Ding WC, et al. has high sensitivity, which can detect 10 copies of ISKNV, and its sensitivity surpasses that of traditional LAMP and standard PCR by factors of 10 and 1000, respectively [52]. The underlying reason for this result may lie in the employment of an increased number of components, which are instrumental in promoting the LAMP reaction. Additionally, this method still utilizes a dual-primer system when measuring the minimum detection limit, which could also be a contributing factor. Compared to LAMP-LFD, LAMP-HNB simplifies the detection process by allowing real-time observation of amplification without the need for a separate lateral flow device. Compared to the closed-tube amplification method described in this paper, it is more prone to producing false positives due to contamination, and the accuracy of the result is more dependent on the pore size and structure of the LFD strip membrane. Although all of these methods have lower detection limits than duplex LAMP-HNB, they still rely on expensive machines or complex devices, while the approach outlined in this paper can operate without such requirements.

In this method, *A. hydrophila*, ISKNV and the mixed positive templates were used as positive controls, ddH_2_O was used as negative template and the LAMP reaction was performed using various common pathogenic nucleic acids from mandarin fish. The results showed that the method established in this paper did not cross-react with other pathogens.

The duplex LAMP-HNB method only requires a common heating device and can even be reacted by a water bath, and the pre-added hydroxynapthol blue can show a visible sky blue color after positive amplification, which is of great significance for the early work of cultivation, because although it is not known what kind of pathogen it is, the appearance of sky blue can provide a reference for on-site detection and initial screening. After 1 h of enzyme digestion, the pathogen species could be determined according to the size of the band, and the identification of the pathogen could be completed in about 2 h. This method achieves simplified operation and real-time detection on site. Although LAMP offers the advantages of high sensitivity and rapid amplification, it is highly susceptible to residual contamination, which can lead to false-positive results. To address this issue, stringent zoning protocols and multiple negative controls were implemented in this study to minimize the risk of contamination. Future research could focus on further optimizing the experimental workflow, such as integrating microfluidic technology or closed detection systems, to enhance the reliability and applicability of LAMP technology.

## 5. Conclusions

In summary, a method for the detection of *A. hydrophila* and ISKNV co-infection by duplex LAMP-HNB was established in this paper, which has good sensitivity and specificity. The analysis can be completed quickly, and due to the simple method and fast process, it can be used for early detection or preliminary screening of common pathogens in mandarin fish, which is conducive to preventing the spread of the pathogens in the farm.

## Figures and Tables

**Table 1:** LAMP primer sequence of ISKNV.

Primers	Sequence (5′ → 3′)	Target Amplification
MCP F3	GCCCGCAGACAATTCCTTG	211 bp
MCP B3	CCGAGGGGTGTTCTCGTAA	
MCP FIP	TTGCGGTGGGTGACGTTCTTTAGTGCATCTGGACCTCAGGT	
MCP BIP	CGTGCAAAGCAACTACACCGCGGGATTGGTGGCCATCAAAG	

**Table 2:** LAMP primer sequence of *A. hydrophila*.

Primers	Sequence (5′ → 3′)	Target Amplification
*hlyA* F3	GGGGTCGAGGTGAACAAGG	250 bp
*hlyA* B3	AGCTGAGCGGCTGGATGC	
*hlyA* FIP	TTCGACCCGGTAATCCTGGGTGGAGGCGTCGGCCAAGT	
*hlyA* BIP	GCGCCCAGAAGGTGAGTTTCAAGGAGAGCAGGGACTCCG	

**Table 3:** Lamp system for ISKNV.

Components	Content (μL)
MCP F3 (10 μM)	0.5
MCP B3 (10 μM)	0.5
MCP FIP (100 μM)	0.4
MCP BIP (100 μM)	0.4
10 × IsothermalAmp Buffer	2.5
MgSO_4_ (100 mM)	1.5
Hydroxynaphthol blue (1.5 mM)	2
dNTPs mix (10 mM)	3.5
Bst II DNA Polymerase Large Fragment (8 U/μL)	1
DNA template	1
ddH_2_O	11.7

**Table 4:** Lamp system for *A. hydrophila*.

Components	Content (μL)
*hlyA* F3 (10 μM)	0.5
*hlyA* B3 (10 μM)	0.5
*hlyA* FIP (100 μM)	0.4
*hlyA* BIP (100 μM)	0.4
10 × IsothermalAmp Buffer	2.5
MgSO_4_ (100 mM)	1.5
Hydroxynaphthol blue (1.5 mM)	2
dNTPs mix (10 mM)	3.5
Bst II DNA Polymerase Large Fragment (8 U/μL)	1
DNA template	1
ddH_2_O	11.7

## Data Availability

The data presented in this study are available on request from the corresponding author. The data are not publicly available due to privacy or ethical restrictions.

## Supplementary Materials

The following supporting information can be downloaded at: https://www.mdpi.com/article/10.3390/microorganisms13030586/s1, Figure S1. Optimization of the duplex LAMP-HNB. (A): optimization of Mg^2+^ content: M: 100 bp DNA Ladder, 1–6 in that order: Mg^2+^ 6 mM, Mg^2+^ 7 mM, Mg^2+^ 8 mM, Mg^2+^ 9 mM, Mg^2+^ 10 mM, negative control. (B): optimization of dNTP content: M: 100 bp DNA Ladder, 1–6 in that order: dNTP 1.0 mM, dNTP 1.2 mM, dNTP 1.4 mM, dNTP 1.6 mM, dNTP 1.8 mM, negative control. (C): optimization of reaction temperature: M: 100 bp DNA Ladder, 1–7 in that order: 60 °C, 61 °C, 62 °C, 63 °C, 64 °C, 65 °C, negative control. (D): optimization of reaction time: M: 100 bp DNA Ladder, 1–5 in that order: 30 min, 40 min, 50 min, 60 min, negative control. Figure S2. Enzyme digestion of the product at the minimum detection limit. M: 20 bp DNA Ladder, 1: 1 pg, NC: negative control.
