# Supplementary material for: Development of Duplex Loop-Mediated Isothermal Amplification with Hydroxynaphthol Blue for Detection of Infectious Spleen and Kidney Necrosis Virus and Aeromonas hydrophila in Chinese Perch (Siniperca chuatsi)
Source: Microorganisms. 2025 Mar 4;13(3):586. doi: 10.3390/microorganisms13030586 (PMC11946703; doi:10.3390/microorganisms13030586)
Supplement: Supplementary file 1 [file microorganisms-13-00586-s001.zip › microorganisms-3424124-supplementary.pdf]

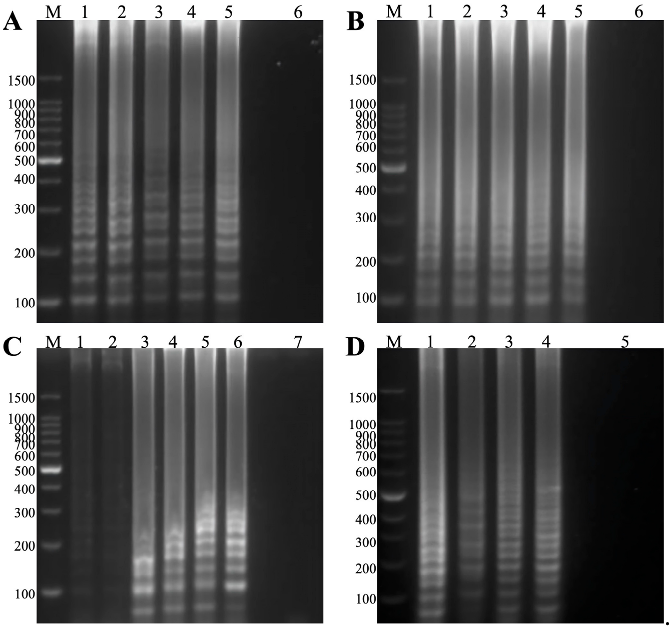

Figure S1. Optimization of the duplex LAMP-HNB. (**A**): optimization of Mg^{2+} content: M:100 bp DNA Ladder, 1–6 in that order: Mg^{2+} 6 mM, Mg^{2+} 7 mM, Mg^{2+} 8 mM, Mg^{2+} 9 mM, Mg^{2+} 10 mM, negative control. (**B**): optimization of dNTP content: M: 100 bp DNA Ladder, 1–6 in that order: dNTP 1.0 mM, dNTP 1.2 mM, dNTP 1.4 mM, dNTP 1.6 mM, dNTP 1.8 mM, negative control. (**C**): optimization of reaction temperature: M: 100 bp DNA Ladder, 1–6 in that order: 60 °C, 61 °C, 62 °C, 63 °C, 64 °C, 65 °C, negative control. (**D**): optimization of reaction time: M: 100bp DNA Ladder, 1–5 in that order: 30 min, 40 min, 50 min, 60 min, negative control.

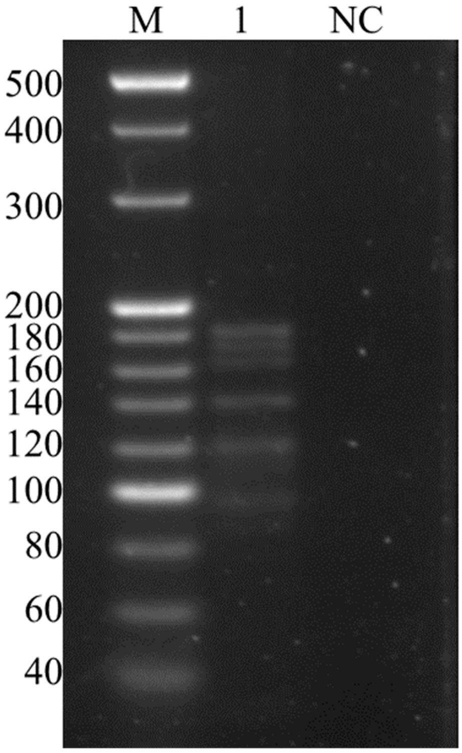

Figure S2. Enzyme digestion of the product at the minimum detection limit. M: 20 bp DNA Ladder, 1: 1 pg, NC: negative control.
